# Supplementary material for: Organic matter lability modifies the vertical structure of methane-related microbial communities in lake sediments
Source: Microbiol Spectr. 2023 Sep 12;11(5):e01955-23. doi: 10.1128/spectrum.01955-23 (PMC10581051; doi:10.1128/spectrum.01955-23)
Supplement: Supplemental methods, tables, and figures — Appendix A: Supplemental methods, Tables S1 and S2, Figures S1 and S2. [file spectrum.01955-23-s0001.doc]

**Appendix A: Supplementary methods, Tables S1 and S2, Figures S1 and S2 of the study:**

**Organic matter lability modifies the vertical structure of methane-related microbial communities in lake sediments**

Antti J Rissanen^a,b*^, Tom Jilbert^c^, Asko Simojoki^d^, Rahul Mangayil^a,#^, Sanni L Aalto^e,f,#^, Ramita Khanongnuch^a^, Sari Peura^g^, Helena Jäntti^e^

^a^Faculty of Engineering and Natural Sciences, Tampere University, Korkeakoulunkatu 6, FI-33720, Tampere, Finland

^b^Natural Resources Institute Finland (Luke), Latokartanonkaari 9, FI-00790, Helsinki, Finland

^c^Environmental Geochemistry group, Department of Geosciences and Geography, Faculty of Science, Gustaff Hällströmin katu 2, 00560 Helsinki, Finland

^d^Department of Agricultural Sciences (Environmental Soil Science), Faculty of Agriculture and Forestry, University of Helsinki, P.O. Box 56, FI-00014, Helsinki, Finland

^e^Department of Environmental and Biological Sciences, University of Eastern Finland, Yliopistonranta 1 E, FI-70210, Kuopio, Finland

^f^Department of Biological and Environmental Sciences, University of Jyväskylä, Survontie 9 C, FI-40014, Jyväskylä, Finland

^g^Department of Forest Mycology and Plant Pathology, Science for Life Laboratory, Swedish University of Agricultural Sciences, Almas allé 5, SE-75651, Uppsala, Sweden

^#^Present address:

Sanni L Aalto: Technical University of Denmark, DTU Aqua, Section for Aquaculture, The North Sea Research Centre, P.O. Box 101, DK-9850, Hirtshals, Denmark

Rahul Mangayil: Department of Bioproducts and Biosystems, School of Chemical Engineering, Aalto University, FI-00076 Aalto, Finland

*Corresponding author: Antti J Rissanen, Faculty of Engineering and Natural Sciences, Tampere University, Korkeakoulunkatu 6, FI-33720, Tampere, Finland. e-mail: [antti.rissanen@tuni.fi](mailto:antti.rissanen@tuni.fi)

**Supplementary methods**

**Bioinformatic analysis of the 16S rRNA gene amplicon reads**

The sequencing service provider, FISABIO, did the quality assessment of the raw data and the merging of the paired-end reads. Quality assessment, i.e., removing of too short and low quality reads, was done using prinseq-lite with the following parameters: min_length: 50; trim_qual_right: 30; trim_qual_type: mean; trim_qual_window: 20 (Schmieder and Edwards 2011). The paired-end reads passing the quality assessment were joined using the FLASH program by applying default parameters (Magoč and Salzberg 2011).

Mothur was used in subsequent sequence analyses (Schloss et al. 2009). The sequences were aligned using Silva reference alignment (Release 132). Chimeric sequences, identified using VSEARCH (Rognes et al. 2016), were removed from each library and a preclustering algorithm (Huse et al. 2010) was used to reduce the effect of sequencing errors. Sequences were assigned taxonomies with a naïve Bayesian classifier (bootstrap cutoff value 80%) (Wang et al. 2007), using the Silva database (Release 132), which was supplemented with a partial 16S rRNA gene sequence from *Candidatus* Methylumidiphilus alinenensis, a novel methanotroph belonging to order *Methylococcales*, which dominated the water column MOB community in a small Finnish boreal lake, Lake Alinen-Mustajärvi (Rissanen et al. 2018). Thereafter, sequences classified as chloroplast, mitochondria and eukaryota were removed from each library. Sequences were clustered into operational taxonomic units (OTUs) at a 97% similarity level. Singleton OTUs (OTUs with only one sequence) were removed, and the data were then normalized by subsampling to the same size, 77340 sequences. One sample, representing the layer 8-9 cm depth at Station 3, was discarded from the analyses since it had only ~10 000 joined sequence reads. Good coverage was 0.95 – 0.97 in each library confirming that sequence variation was adequately covered.

**References**

Huse, S.M., Welch, D.M., Morrison, H.G., et al., 2010. Ironing out the wrinkles in the rare biosphere through improved OTU clustering. Environ. Microbiol. 12, 1889-1898.

Magoč, T., Salzberg, S.L., 2011. FLASH: fast length adjustment of short reads to improve genome assemblies. Bioinformatics 27, 2957-2963.

Rissanen, A.J., Saarenheimo, J., Tiirola, M., et al., 2018. Gammaproteobacterial methanotrophs dominate methanotrophy in aerobic and anaerobic layers of boreal lake waters. Aquat. Microb. Ecol. 81, 257-276.

Rognes, T., Flouri, T., Nichols, B., et al., 2016. VSEARCH: a versatile open source tool for metagenomics. PeerJ 4, e2584.

Schloss, P.D., Westcott, S.L., Ryabin, T., et al., 2009. Introducing mothur: open-source, platform-independent, community-supported software for describing and comparing microbial communities. Appl. Environ. Microbiol. 75, 7537-7541.

Schmieder, R., Edwards, R., 2011. Quality control and preprocessing of metagenomic datasets. Bioinformatics 27, 863-864.

Wang, Q., Garrity, G.M., Tiedje, J.M., et al., 2007. Naïve Bayesian classifier for rapid assignment of rRNA sequences into the new bacterial taxonomy. Appl. Environ. Microbiol. 73, 5261-5267.

**Table S1** The detailed statistics of regression models shown in Fig 3.

**Table S2** Spearman correlation analysis results^a^ on the relationship between microbial variables (excluding *Methanomassiliicoccales* from the total methanogens) and environmental variables indicating sediment OM lability, i.e., C:N ratio^b^ of the surface sediments (representing C:N ratio of sedimenting OM) and concentration of porewater dissolved inorganic carbon (DIC)^c^.

| **Microbial variables** | **C:N^b^** | **C:N^b^** | **DIC^c^** | **DIC^c^** |
| --- | --- | --- | --- | --- |
|  | **(aver.)** | **(max.)** | **(aver.)** | **(max.)** |
| Relative abundances: |  |  |  |  |
| Total methanogens except *Methanomassiliicoccales* (4-10 cm) | **-0.9** | **-0.9** | **0.9** | **0.9** |
| Total Methanogens except *Methanomassiliicoccales* (0-10 cm) | **-0.9** | **-0.9** | **0.9** | **0.9** |
| Ratios of relative abundances: |  |  |  |  |
| Total methanotrophs (0-3 cm)/Total methanogens except *Methanomassiliicoccales* (4-10 cm) | **0.9** | **0.9** | **-0.9** | **-0.9** |
| *Ca*. Methylomirabilis (0-3 cm)/Total methanogens except *Methanomassiliicoccales* (4-10 cm) | **0.9** | **1** | **-0.9** | **-1** |
| *Methylococcales* (0-3 cm)/Total methanogens except *Methanomassiliicoccales* (4-10 cm) | 0.8 | **0.9** | -0.8 | **-0.9** |
| Total methanotrophs (0-3 cm)/Total methanogens except *Methanomassiliicoccales* (0-10 cm) | **0.9** | **0.9** | **-0.9** | **-0.9** |
| *Ca*. Methylomirabilis (0-3 cm)/Total methanogens except *Methanomassiliicoccales* (0-10 cm) | **0.9** | **1** | **-0.9** | **-1** |
| *Methylococcales* (0-3 cm)/Total methanogens except *Methanomassiliicoccales* (0-10 cm) | 0.8 | 0.8 | -0.8 | -0.8 |

^a^n=5, statistically significant results (p < 0.05) highlighted in bold. Negative and positive correlations with C:N ratio and positive and negative correlation with DIC indicate increase and decrease, respectively, with increasing sediment OM lability. Aver. and max in brackets below the column title denote whether average or maximum relative abundance, respectively, in 0-3 cm (for methanotrophs), 4-10 cm or 0-10 cm sediment layers (for methanogens) was used in the analysis (see text).

^b^Using either C:N ratio in 0-1 cm sediment layer or C:N ratio of 0-2 cm sediment layer (i.e., average C:N ratio of 0-1 cm and 1-2 cm sediment layers) gave identical results. The C:N ratios are shown in Table 1.

^c^Average porewater DIC concentration within the 0-10 cm sediment layer. The values are shown in Table 1.

Fig. S1

**Fig. S1.** Vertical profiles of temperature (left panels) and dissolved O_2_ (DO) (right panels) in the water columns at the five study stations during the sediment sampling.

**Fig. S2**

**Fig. S2.** Vertical variation in A) C:N ratios of the sediment, B) concentrations of dissolved inorganic carbon (DIC) and C) acetate and lactate (lactate detected only in St 1) in the sediment porewaters (and water above the sediment), as well as D) Shannon diversity index of prokaryotic diversity (assessed via 16S rRNA gene amplicon sequencing) in sediment, at the 5 study stations. Depth 0 cm (dashed line in B and C) indicates sediment-water interface.
